# Stable Reference Gene Selection for RT-qPCR Analysis in* Synechococcus elongatus* PCC 7942 under Abiotic Stresses

**DOI:** 10.1155/2019/7630601

**Published:** 2019-04-21

**Authors:** Xiao Luo, Jiaxing Li, Tianliang Chang, Hongyan He, Yi Zhao, Xue Yang, Yuwei Zhao, Yao Xu

**Affiliations:** ^1^Provincial Key Laboratory of Biotechnology of Shaanxi Province, Northwest University, Xi'an 710000, China; ^2^College of Life Sciences, Northwest University, Xi'an 710000, China; ^3^Key Laboratory of Resource Biology and Biotechnology in Western China (Ministry of Education), Northwest University, Xi'an 710000, China

## Abstract

*Synechococcus elongatus* PCC 7942 (*S. elongatus *PCC 7942) is a model cyanobacteria species for circadian clock mechanism studies. It has also been widely used as a bioreactor to produce biofuels and other metabolic products. Quantitative real-time PCR (qPCR) technology is the most commonly used method for studying the expression of specific genes, in which the relative expression level of target genes is calibrated by stably expressed internal reference genes. In this work, we examined the expression of nine candidate reference genes in time-course samples of* S. elongatus* PCC 7942 under no treatment (control), NaCl-stress conditions, H_2_O_2_-stress conditions, and high light-stress conditions. Based on the qPCR amplification parameters, the stability ranking of these candidate reference genes was established by three statistical software programs, geNorm, NormFinder, and BestKeeper. Considering all the stress conditions or high light stress alone, the results showed that the combination of* prs *and* secA* was the best choice for the double reference gene calibration method by qPCR. The combination of* secA* and* ppc*,* rimM* and* rnpA*,* rnpA,* and* ilvD* was most stable under no treatment, NaCl-stress conditions, and H_2_O_2_-stress conditions, respectively.* rimM* was stable under only special conditions and should be carefully chosen.* 16S* and* rnpB* were not suitable as internal reference genes for* S. elongatus *PCC 7942 qPCR experiments under all experimental conditions. To validate the above results, a cyanobacterial core clock gene,* kaiC*, was used to evaluate the actual performance of the optimized reference genes by qPCR, as well as the worst reference genes under different stress conditions. The results indicated that the best reference gene yielded more accurate calibration results for qPCR experiments carried out in* S. elongatus *PCC 7942 time-course samples.

## 1. Introduction

*Synechococcus elongatus* PCC 7942 (*S. elongatus* PCC 7942) is a freshwater single cell photosynthetic autotrophic prokaryote [1, 2]. Its 2.74 Mb genome constitutes 2,715 genes and encodes approximately 2,612 proteins [3].* S. elongatus *PCC 7942 is also the simplest model organism for studying circadian rhythms [4, 5], with approximately 30% of its genes showing circadian rhythmic expression profiles that peak at dawn or dusk [6, 7]. The cyanobacterial clock is controlled by genetic, physiological, and metabolic processes, such as cell division, nitrogen fixation, photosynthesis, amino acid uptake, carbohydrate synthesis, and respiration [8, 9]. The central oscillator of* S. elongatus* PCC 7942 is composed by three clock proteins, KaiA, KaiB, and KaiC [10]. Hexameric KaiC proteins, the key components of the* S. elongatus* PCC 7942 clock, have autokinase, phosphotransferase, and ATPase activities and display a circadian rhythmic oscillation of phosphorylation and dephosphorylation every day [11]. Interestingly, circadian rhythmic oscillations due to KaiC phosphorylation can be reconstituted in vitro when purified KaiA, KaiB, KaiC proteins and an appropriate amount of ATP are incubated together [12]. During the growth of cyanobacteria, the most common stresses come from various environmental abiotic factors, such as salinity [13], light stress [14], and accumulated peroxide [15]. To understand the effects of different types of stress on cyanobacteria, it is necessary to evaluate the expression patterns of cyanobacteria exposed to different stress conditions.

Quantitative real-time PCR (qPCR) technology can be used to monitor the production and accumulation of amplification products in each cycle of the PCR reaction by adding a fluorescent dye or a fluorescent probe to the PCR reaction. This method is characterized by its simplicity, sensitivity, high specificity and high throughput features. qPCR has become a basic and standard technique for detecting or comparing the level of a defined gene in a given experimental condition [16, 17]. However, many parameters in a qPCR experiment can affect the authenticity of the experimental results, such as the quality of the sample RNA, the efficiency of the reverse transcription reaction, and the stability of the internal reference gene. Standardized qPCR requires the elimination of systematic errors between different sample groups as much as possible, so that the final experimental results reveal the closest biological changes [18]. This makes it particularly important to reduce the variability of the experimental parameters and to enhance the stability of the experimental setup when using large-scale sample populations for analyzing specific gene expression levels, such as using the qPCR technique, to perform rhythmic expression detection of defined gene populations in time-course samples. The internal reference gene used to calibrate the expression of the target gene is one of the key technical means for achieving qPCR standardization and increasing the stability of the experimental system. Although commonly used internal reference genes are usually the so-called housekeeping genes necessary to maintain cell survival [19], the results of earlier studies show that the expression levels of housekeeping genes under different experimental treatments and environmental conditions significantly fluctuate [20]. When calibrating the expression level of a target gene, the use of relatively unstable housekeeping genes may lead to misinterpretation of the real results [21]. Therefore, when qPCR technology is used, not only the appropriate biological repeats should be set, the quality of RNA samples and reverse transcription synthesis cDNA templates should be strictly controlled, and the optimal internal reference genes under experimental conditions should be carefully screened [22, 23].

The prescreening of housekeeping genes, which will be used as internal reference genes in qPCR, is mainly carried out in studies of eukaryotes. Except for a few reports in the cyanobacteria* Synechocystis *sp. PCC 6803 [24] and* Synechococcus *sp. PCC 7002 [25], the screening of qPCR internal reference gene in other prokaryotes, including* S. elongatus* PCC 7942, has not been reported. When using qPCR for transcriptional studies in cyanobacteria, the most commonly used internal reference gene encoding is 16s RNA (16S ribosomal RNA) because it is highly conserved during evolution [26–29]. In addition,* rnpA* [24, 30],* rnpB *[24, 31–33],* ppc* [34],* ilvD* [24],* secA* [24, 25],* petB *[24, 25, 30],* rim* [25], and* prs* [24] are also commonly used internal reference genes in cyanobacterial transcriptome research. In recent years, various algorithms for analyzing the stability of the expression levels of housekeeping genes have been developed. Currently, there are various free software packages that are available online, such as geNorm [35], NormFinder [36], BestKeeper [37], ΔCt approach [38], and RefFinder [39]. Based on the Ct values of the qPCR reaction from individual candidate reference genes, the corresponding stability evaluation is given according to a similar algorithm, though different software packages designate different weight values to the core parameters, such as the Ct value, amplification efficiency, and intergroup difference of the qPCR reaction. The final ranking results of the candidate reference genes could also be inconsistent between different software programs. Therefore, it is necessary to use two or more algorithms simultaneously to identify the most stably expressed reference gene [39]. Our current study used RT-PCR to detect the expression patterns of nine candidate reference genes (shown in Table 1) under NaCl, high light, and H_2_O_2_ conditions in the cyanobacteria* S. elongatus* PCC 7942. Three kinds of software, namely, geNorm, NormFinder, and BestKeeper, were used to evaluate the stability of these candidate internal reference genes for qPCR experiments under various stress conditions. Finally, we used a core circadian clock gene* kaiC *[40, 41] to verify the optimal reference genes screened by the software programs. The relevant research results provide an experimental basis for further study of gene expression analysis in* S. elongatus* PCC 7942 under stress conditions.

## 2. Materials and Methods

### 2.1. Strains, Culture Conditions, and Sample Collection

Wild-type* S. elongatus* PCC 7942 (R^2^ strain) was purchased from the Wuhan Institute of Aquatic Biology, Chinese Academy of Sciences. The* S. elongatus* PCC 7942 cells were cultured in 150 ml fresh BG11 (Haibo, Qingdao, China) liquid medium with gentle shaking (100 rpm) at 30°C under 50 *μ*E•m^−2^•s^−1^ continuous light provided by cool-white fluorescent bulbs. When the OD_750_ of cyanobacteria cultures reached approximately 0.3, the cultures were divided into four groups, namely, no treatment control (NTC), NaCl-treated group (NaCl), H_2_O_2_-treated group (HO), and high light intensity-treated group (HL). Three biological replicates were set up in each group. After being entrained with two 12-h light/12-h dark cycles at 30°C, the cyanobacteria cultures were treated with 1% NaCl (w/v) or 1 mM H_2_O_2_ in fresh liquid BG11 medium for 48 h. For the HL group, the entrained cyanobacteria cells were cultured under 300 *μ*E•m^−2^•s^−1^ for 48 h after entrainment. Subsequently, 10 ml of the cyanobacteria cell suspension from each group was centrifuged at 4,500 rpm for 15 min and stored in a −80°C freezer for the following experiments. Time-course samples from NTC, NaCl, HO, and HL groups were continually collected every 4 h for 48 h.

### 2.2. Total RNA Extraction and cDNA Synthesis

The frozen time-course cyanobacteria samples were resuspended and incubated in 100 *μ*L of lysozyme solution (RNase-free, Sangon, Shanghai, China) for 10 min at RT, according to the manufacturer's instructions. Total RNA from the digested samples was isolated using a Bacterial Total RNA Extraction and Purification Kit (Sangon). To detect the quality total RNA samples, all RNA samples were detected by 1.5% agarose gel electrophoresis. As shown in Figure S1, the total RNA samples obtained by the method reported in these work were qualified to be the templates for following reverse transcription and qPCR reactions. The A_260_/A_280_ ratios of total RNA samples varied from 2.0 to 2.1, while their A_260_/A_230_ ratios were around 1.8 (as shown in Figure S2). All these data indicated that the quality of total RNAs was guaranteed and could completely meet the requirement of the following qPCR experiments. The total RNA samples were simultaneously treated with 1 *μ*L/mL Dnase I (Rnase-free, Takara, Dalian, China) and RNase Inhibitor (Takara). The total RNA samples were diluted to a final concentration of 1.0 *μ*g/*μ*L and used as templates for cDNA synthesis using the PrimeScript™ II First Strand cDNA Synthesis Kit (TaKaRa), according to the manufacturer's instructions. A universal DNA Purification Kit (TIANGEN, China) was used to purify cDNA products.

### 2.3. Primer Design and Amplification Characteristics of Candidate Reference Genes

Nine candidate reference genes from various species of cyanobacteria were selected from previous reports. The sequences of the homologous internal reference genes were downloaded from the database CYORF http://cyano.genome.jp/. The detailed information for these candidate reference genes is shown in Table 1. Software Primer Premier (version 5.0) was used to design the primers for qPCR amplification. All primers were designed to meet the following standards: a melting temperature (Tm) between 50 and 65°C, a primer length between 19 and 25 bp, a product length between 100 and 300 bp, and a GC content of 50–60%. The specificity of each primer pair was tested via semiquantitative RT-PCR using cDNA from NTC samples as the templates, whereas the Premix Taq™ Kit (TaKaRa) was used to catalyze the amplification reactions. The semiquantitative RT-PCR amplified product for each candidate gene was separated on 1% agarose gel and visualized using a GelDoc XR System (Bio-Rad, Hercules, CA, USA).

### 2.4. The Performance of qPCR

**Figure 1:**
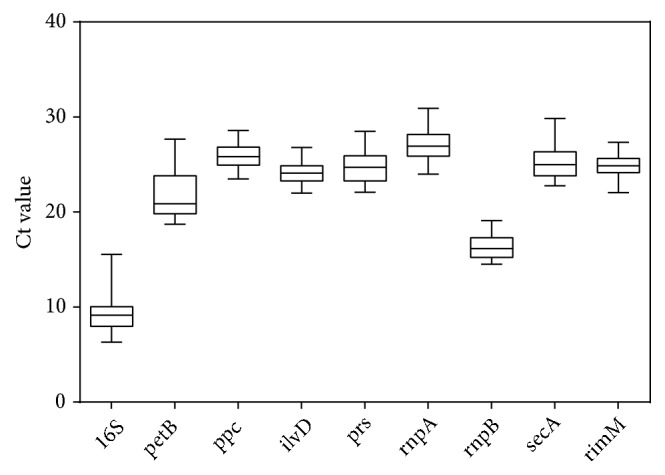
Range of cycle threshold (Ct) values of the nine candidate reference genes across all samples of* S. elongatus* PCC 7942. The box indicates the Ct value within the 25th and 75th percentiles. Whiskers include the Ct values in the 10th and 90th percentiles. The line across the box represents the median.

qPCR was carried out on a Bio-Rad CFX96 Real-time PCR System (Bio-Rad), according to the manufacturer's instructions. The 10 *μ*L reaction was composed of 5 *μ*L of 2×SYBR Premix Ex TaqII (TaKaRa), 0.25 *μ*L (20 *μ*M) of each upstream primer and downstream primer, 3.5 *μ*L of ddH_2_O, and 1 *μ*L of appropriately diluted cDNA template. A two-step RT-qPCR was performed using the following protocol: predenaturation at 95°C for 3 minutes, followed by denaturation at 95°C for 10 seconds and annealing at the appropriate temperature for 30 seconds (the individual optimized annealing temperature for the different candidate reference genes is listed in Table 1), which was repeated for 40 cycles. To confirm the specificity of the primers, melting curve analysis was carried out during the heating phase from the individual optimized annealing temperatures to 95°C at the end of each cycle. Three replicates were set for the cDNA of each biological sample, and the final Ct value of each individual experiment was counted by the average of three independent biological replicates from different batches of experiment, whereas each biological replicate was represented by an average of three parallel technical replicates from the same cDNA templates. The Ct values from all samples were uploaded to GraphPad Prism v6.04 Software to generate box-and-whisker plots, which could indicate the Ct value within the 25th and 75th percentiles (Figure 1). Whiskers of each sample covered the Ct values in the 10th and 90th percentiles, whereas a horizontal solid line in the box represented the median.

The E value corresponded to the efficiency of a given qPCR amplification [22]. To determine the E value of a qPCR reaction, we prepared a 1:10 serial dilution of the original cDNA template (diluted from 10^−2^ to 10^−8^). Subsequently, each candidate gene was amplified using the diluted cDNA template using standard qPCR. The E value and the regression coefficient (R^2^) of qPCRs were calculated according to the dilution gradients and the Ct values obtained from the individual qPCRs. The E value was calculated by the following equation: E = [10^-(1/slope)^-1]*∗*100% (Table 1). Uniformly spaced amplification curves produced a linear standard curve with a reaction efficiency of 90–110% for qPCR [22].

### 2.5. Analysis of Reference Gene Expression Stability

The reference gene stability analysis software programs geNorm (version 3.5), NormFinder (version 0.953), and BestKeeper (version 1.0) were used to analyze the performance of the candidate housekeeping genes under different stress conditions. The geNorm software determined the two best internal reference genes by determining their stability values, represented as M values. The M value of each candidate reference gene was calculated by comparing the average standard deviation between the log-transformed expression ratios of the target genes to that of another candidate gene [42]. The reference gene with the lowest M value was rejected for further analysis. This comparison process between the candidates was repeated until the two most stable reference genes were identified [22]. An M value of 1.5 was used as a criterion for evaluating the stability of a tested reference gene. When the M value of a candidate was lower than 1.5, it was considered an internal reference gene. When the M value was higher than 1.5, the candidate gene was excluded. In addition, the pairwise variation comparisons between two consecutive normalization factors (V_n_/V_n+1_) determined the number of optimal internal reference genes that were needed in the qPCR, with 0.15 set as a threshold. When the value of V_n_/V_n+1_ was lower than 0.15, it indicates that less than n additional reference genes were necessary for the following target gene calibration processes [35]. NormFinder was used to assess the stability of the reference gene expression based on the intra- and intergroup variations among the candidates. With a similar criteria to geNorm but calculated by different strategies, NormFinder chose the gene with lowest standard deviation as the most stable reference gene [43], that is, the gene with the lowest M value was considered as the most stable one. BestKeeper is a Microsoft Excel based tool for identifying the most suitable reference gene by pairwise correlation analysis among the candidates [37]. Descriptive statistical results of Ct values of all reference genes under different experimental conditions, including standard deviation (SD) and variance coefficient (CV), were calculated. The Pearson correlation coefficient, designated as r, for each candidate internal reference gene pair was calculated. A highly correlated candidate reference gene with a low standard deviation (SD < 1) was combined into the index value (i.e., a normalization factor) using a geometric mean of its Ct values, and each candidate reference gene and index value were determined by BestKeeper software (the correlation coefficient r value was defined as the BestKeeper Index), and the probability (P) value. The gene having the highest r value and a p-value below 0.05 was considered to be the most stable.

## 3. Results

### 3.1. The Candidate Reference Genes for qPCR

Nine common reference genes in cyanobacteria,* 16S*,* petB*,* ppc*,* ilvD*,* prs*,* rnpA*,* rnpB*,* secA,* and* rimM*, were selected to be evaluated as candidate references for the analysis of gene expression under stress conditions (NaCl, H_2_O_2_, and high light stress). The applicability of the primers designed for the qPCR amplification of these nine candidate genes were verified by RT-PCR and the sequencing of the amplified products (Figure S3). Subsequently, the specificity of the qualified primers was tested by unimodal melting curve analysis (Figure S4). The information of all nine candidates, such as their gene IDs, gene definitions, PCR primer sequences, size of the PCR products, Tm values, amplification efficiencies, correlation coefficients (R^2^), and median Ct values, is listed in Table 1. The data for KaiC, the target gene for verifying of candidate references, is listed in Table S1. In general, the sizes of the qPCR products of all the tested genes ranged from 102 bp to 296 bp, with efficiency E values ranging from 90.4% to 105.1%, and the R^2^ distributing from 0.996 to 0.999. As shown in the box-and-whisker plot in Figure 1, the distribution of the Ct values from the nine reference genes under different conditions varied from 6.31 to 30.91. The* 16S* RNA gene encoded the most abundant transcripts in cyanobacteria* S. elongatus *PCC 7942, and the* 16S* RNA had the lowest median Ct value (Ct<10, Table 1) compared to other candidate reference genes during qPCR amplification when the same amounts of total RNA were used as templates for reverse transcription reactions.

### 3.2. Analysis Based on geNorm Software

**Figure 2:**
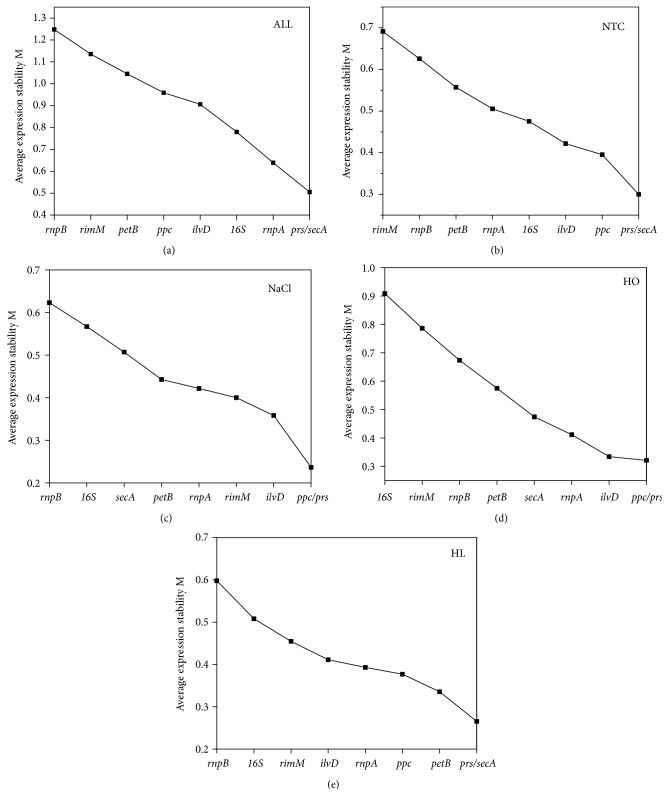
Gene expression stability M value and ranking of the nine candidate reference genes as calculated by geNorm software. (a) All samples. (b) No treatment Control (NTC). (c) NaCl stress (NaCl). (d) H_2_O_2_ stress (HO). (e) High light stress (HL). The stability of the gene increased from left to right.

Processed by geNorm software, the M values of all the candidate reference genes were used to determine the rank of their expressing stabilities. The threshold of the M value was set at 1.5. A smaller M value was indicative of more stable gene expression. However, M values higher than 1.5 caused the rejection of the candidate to be a qualified internal reference gene. According to result of the geNorm statistical analysis, the M values of all the candidate internal reference genes were lower than 1.5 under all the tested experimental conditions (Figure 2). When the data from the four tested conditions were counted,* secA* and* prs* had the lowest M value of 0.505 and were the most stably expressed candidate genes. Furthermore, the M values of 0.300 and 0.265 under NTC and HL, the lowest score among all the candidates, rendered* secA* and* prs* as the most suitable reference genes under these situations. When NaCl and HO were the stress factors,* prs* and* ppc* (M under NaCl=0.237 and M under HO=0.321) were the most stable reference genes by geNorm software. Therefore, either under individual experimental conditions or all four conditions,* prs *was the most stably expressed candidate reference gene suggested by geNorm software.* rnpB *was the most unstable candidate under NaCl or HL, and* rimM* was the most unstable candidate under NTC, whereas* 16S* was the most unstable under HO.

**Figure 3:**
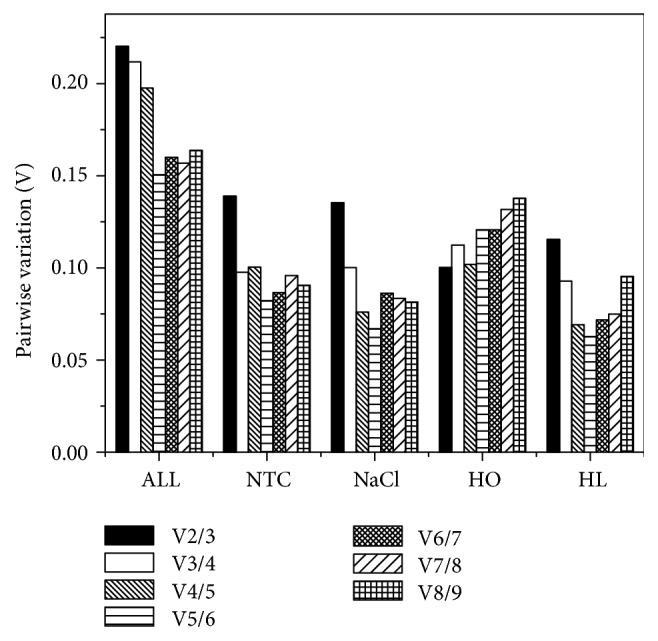
The pairwise variation (Vn/n+1) of the nine candidate reference genes under various test conditions calculated by geNorm software, and the number of optimal reference genes was determined by a threshold of 0.15.

Additionally, geNorm software can also estimate the minimal numbers of the necessary reference genes in a reliable qPCR reaction by a pairwise variation comparison between the reference genes (V_n_/V_n+1_). If the V_n_/V_n+1_ was less than 0.15, it indicated that the using of n+1 reference genes to calibrate the relative expression of target genes was unnecessary. As shown in Figure 3, under NTC, NaCl, HO, and HL conditions, the V_2/3_ values were all below 0.15 (0.1390, 0.1353, 0.1002, and 0.1154, respectively), indicating that the combined use of two reference genes was necessary for qPCR experiments in cyanobacteria under all tested stress conditions. In this study, the best reference gene pairs under NTC and HL conditions given by geNorm were* secA* and* prs*. Under NaCl and HO stress, the best gene pairs are* prs* and* ppc.*

### 3.3. Analysis Based on NormFinder Software

When the expression stability of candidate reference genes was evaluated by NormFinder analysis, a lower stability value was indicative of a more stably expressed reference gene. The ranking results of the candidate genes suggested by the NormFinder are shown in Table 2. When all data from the four experimental groups were evaluated comprehensively,* prs *and* secA *were the most stable genes, which was consistent with the results obtained by geNorm software. When the NTC condition was considered,* secA* and* ppc* were the most stable reference genes. By contrast, the most stably expressed reference genes were* rimM* and* ilvD* under NaCl and* rnpA* and* ilvD *under HO stress. In regard to HL stress,* rnpA* and* prs *were the most stable candidates.* rnpB* displayed the most unstable expression in either NaCl or HL groups, though* 16S* is the most unstable candidate under HO stress. In the NTC group,* rimM* was the most unstable gene under NTC conditions, but at the same time this gene was the most stable under NaCl conditions. This contradiction indicated that the stable expression of a so-called housekeeping gene under normal growth conditions, such as* rimM,* could be severely changed by environmental stresses such as salt in this work.

Under the three stress conditions,* rimM*,* ilvD,* and* rnpA *shared much higher ranks arranged by NormFinder than that obtained via geNorm analysis. A possible reason for these differences between these two sets of software can be ascribed to their different build-in algorithms and weighting methods to the same row data based on the Ct value from the qPCR amplifications. In summary, similar ranking results of the candidate genes were obtained by NormFinder and geNorm software.

### 3.4. Analysis Based on BestKeeper Software

BestKeeper is another popular software, which has been used to optimize the reference genes in qPCR. Unlike the aforementioned software programs, BestKeeper uses a unique indicator, designated as the BestKeeper Index, to evaluate the stability of all candidate reference genes [37]. In this work, the BestKeeper Index performances of the candidate reference genes suggested by the BestKeeper software analysis are ranked and listed in Table 3. From the combined results of all the experimental groups, the top five candidate genes in the list, namely,* secA*,* prs*,* 16S*,* petB,* and* rnpA* showed strong correlation (r>0.92), and the P-values of these candidates were 0.001. When only the NTC condition was concerned,* 16S* and* secA* showed strong correlations with BestKeeper Index (r>0.90). Under NaCl stress, only* petB* had r>0.90. However, under HO and HL stress, the top three genes had a very strong correlation with the BestKeeper Index (r>0.95). A high r value indicated that these candidate genes exhibited highly similar stable expression patterns. In contrast, the r value of* rnpB* in each group (0.477<r<0.745) was much lower. In addition, the p-value (0.003<p<0.099) was higher than that for the other candidate genes. Thus,* rnpB* was considered to be the least reliable reference gene under the preset conditions of the present study in* S. elongatus* PCC 7942. The performance of* rimM* varied dramatically under the different conditions, which was verified to be one of the most unstable candidate genes under NTC stress (r=0.331, p=0.271) and HO stress (r = 0.506, p = 0.077), but also was ranked into the top three confident references under NaCl stress (r = 0.882, p = 0.001).

### 3.5. Comprehensive Stability Analysis of Candidate Reference Genes

Due to the fact that the results from the three software programs were inconsistent, we conducted a comprehensive assessment of all the analyzed data by rearranging the order of the nine candidate reference genes based on the basic results of geNorm, NormFinder, and BestKeeper evaluation. Firstly, the optimal number of essential reference genes in a qPCR reaction was determined to be two, according to the geNorm analysis shown in Tables 4 and 5. In regard to the data integrated from all the four tested conditions or the data under HL stress alone,* prs* and* secA* were the most stable expressed reference genes. Under NTC conditions,* secA* and* ppc* were the most stable genes. In terms of the NaCl group,* rimM *and* rnpA *were the best reference genes, whereas under HO conditions,* rnpA* and* ilvD *were considered to be the best. On the other hand,* rimM* was the most unstable gene under NTC and HO conditions, whereas* rnpB* was found to be the most unstable expressed candidate gene under NaCl and HL.

### 3.6. Validation Test of the Chosen Reference Genes

To verify the validation and applicability of the optimized reference genes suggested by the three software programs, we used the well-studied cyanobacterial clock gene* kaiC* to evaluate the three sets of reference genes, the best reference gene combination (as recommended by geNorm software), and the use of the best or the worst reference gene. Due to the fact that* rimM* was the best reference gene under NaCl stressed condition and was unstable under the other tested experimental conditions (Table 4), we chose the second best gene,* rnpA*, as the optimal reference gene. As shown in Table 4, under all experimental conditions, the circadian expression of* kaiC* peaked near the subjective dusk (approximately 9-12h), regardless of whether the optimal reference gene combination or a single optimal reference gene had been used to calibrate the Ct value of the qPCR amplification. This was consistent with a previous report on the circadian expression profile of* kaiC* under constant lighting conditions [10]. Interestingly, the single use of* rnpA* to calibrate the expression of a target gene under HO showed better performance in qPCR than the use of* rnpA* and* ilvD* in combination as geNorm software had recommended.

When calibrated by* rimM*, the rejected candidate reference gene under NTC stress, the diurnal peak of* kaiC* was significantly deviated from its reported trend. When the rejected candidates were used as internal references in the qPCR experiments, the peak time of the target gene not only was seriously affected, and even caused a reversed phase to the results similar to* rimM* under HO stress, but also resulted in some misleading findings, as the usage of* rnpB *displayed an incorrect diurnal double-peaks phenotype of* kaiC* expression under NaCl stress. Similar results were also found under HO conditions. When the rejected reference gene,* rnpB*, was used to calibrate the relative expression of* kaiC* by qPCR, the amplitude of the target gene diurnal expression profiles was significant dampened, and the SD values between samples were enhanced than those when the suggested best reference gene or the best reference combination was used to calibrate the same qPCR data. Therefore, the results of the rhythmic expression of* kaiC* by different candidate reference genes have indicated that the best reference gene or best reference combination recommended by geNorm, NormFinder, and BestKeeper software can be applied convincingly in qPCR reactions under NaCl, HO, or HL stress conditions in cyanobacterium* S. elongatus *PCC 7942.

## 4. Discussion

When the expression of the target gene is analyzed at the level of RNA transcription, there are inevitable operational errors due to the rapid extraction of large-scale biological samples and the subsequent preparation and quantification of the RNA samples. In addition, the absolute expression level of the same target gene can display significant errors between different biological groups or technical repetitions among different sample groups, which always leads to inaccurate biometric analysis. To address this issue, researchers often select one or more appropriate stably expressed genes, the so-called “housekeeping” genes as internal references. First, the absolute expression level of the target gene is calibrated based on the expression level of the internal reference gene. Thereafter, the normalized relative expression level of the target gene is used as an indicator to objectively describe the differential expression of the target gene under different experimental conditions or at different developmental stages [44]. qPCR is a common technique used for differential expression analysis under various experimental conditions for limited-scale closed gene populations at the RNA level. It has been reported that the selection of different reference genes significantly affects the acquisition of convincing qPCR results [45]. Using inappropriate reference genes for raw data calibration results in significant bias in the final results [46]. In this study, nine housekeeping genes in cyanobacteria* S. elongatus* PCC 7942 were selected as candidate reference genes for studies that evaluated the expression stability in cyanobacteria time-course RNA samples by qPCR that were held under different experimental conditions. To avoid bias in the experimental results caused by the use of a single evaluation software and a single algorithm, we selected three mainstream gene expression stability evaluation software programs based on different algorithms, namely, geNorm, NormFinder, and BestKeeper. The expression stability of selected nine candidate reference genes was detected and evaluated using the qPCR method. Based on the analysis of the three software programs, the expression stability of these candidate reference genes was comprehensively arranged, and the best reference gene of* S. elongatus* PCC 7942 under different experimental conditions was selected.

Pinto et al. reported that* secA* is the most suitable reference gene in the expression analysis of cyanobacteria* Lyngbya aestuarii* CCY 9616 and can be widely used in the calibration of the cyanobacteria qPCR amplification data [24]. In addition, Szekeres et al. showed in cyanobacteria* Synechococcus *sp. PCC 7002 that* secA* is stable under the given experimental conditions and is the best reference for RT-qPCR experiments [25]. These findings are consistent with the combination of the best reference genes we screened under NTC stress. Our results indicated that the* secA *gene could be stably expressed in different species of cyanobacteria under nonstress conditions. It is the optimal choice as the universal reference gene of cyanobacteria in qPCR experiments in the absence of environmental stress. In previous studies, the* rnpA* gene encoding the protein subunit of ribonuclease P (RNase P) was identified in various species of cyanobacteria, such as* Synechocystis *sp. PCC 6803,* Lyngbya aestuarii *CCY 9616,* Nostoc *sp. PCC 7120, and* Synechococcus *sp. PCC 7002, and it is the best internal reference gene [24, 25, 47]. Likewise, our results showed that in* S. elongatus* PCC 7942,* rnpA* was the most stable gene under HO stress. The* ilvD* gene ranks second in stability under HO stress, which is similar to a previous study that reported this gene obtained from* Nostoc *sp. PCC 7120 could serve as an optimal reference gene under specific experimental conditions [24].

**Figure 4:**
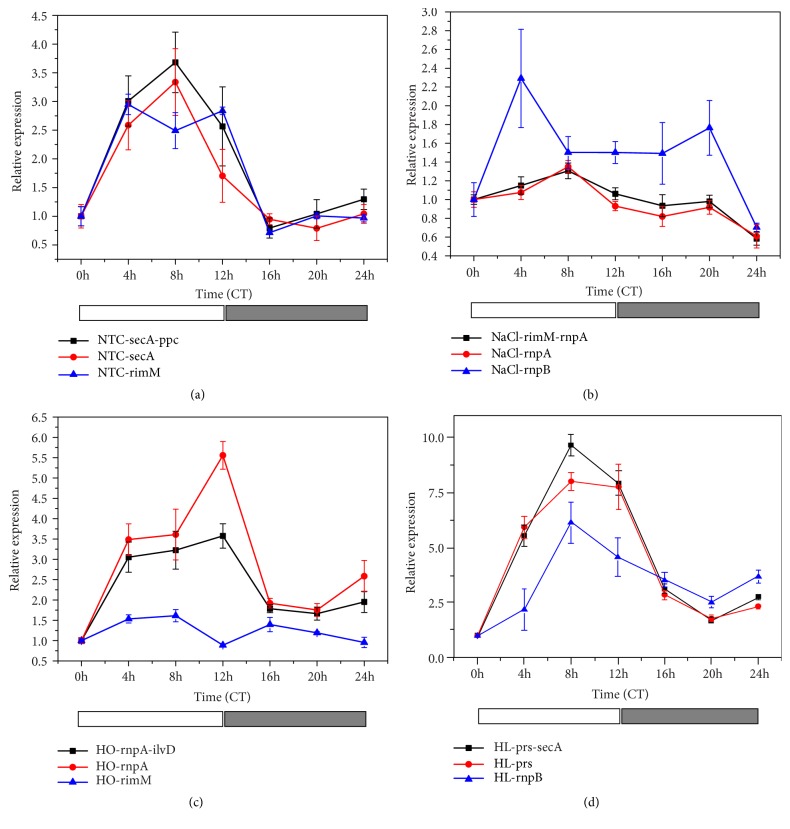
The relative expression level of the cyanobacterial core clock gene* kaiC* under different experimental conditions was normalized to the most stable paired reference genes, the most stable single reference gene, and the most unstable reference gene. Templates were collected for 0–24 h under each treatment condition. (a) NTC. (b) NaCl. (c) HO. (d) HL. Bars represent the standard error from three biological replicates. The white bars under the X-axis indicate CT_0-12_, the subjective daytime, and the gray bars under the X-axis indicate CT_12-24_, the subjective night time.

Surprising results were found in the stability analysis of transcripts amount of the* rimM* gene that encodes the 16S rRNA processing protein. Although the* rimM* gene ranked 8th in the comprehensive ranking of the nine candidate reference genes under the four experimental conditions, it was considered to be the worst qPCR reference gene under NTC and HO conditions. However, the expression stability of this gene ranked first under NaCl stress. RNA microarray analysis carried out in* Synechocystis *sp. PCC 6803 showed that salt stress from the environment strongly induced the expression of genes encoding ribosomal proteins [48]. Therefore, we hypothesized that NaCl treatment may have affected the processing and assembling of the ribosome in* S. elongatus *PCC 7942, so that the expression of* rimM* gene may have been involved in the regulation of cells, where it was stably expressed. Before Szekeres's report in 2014,* rimM* has not been reported as a reference gene for cyanobacterial gene expression studies. Based on the performance of the* rimM *gene in that study, Szekeres et al. suggested that* rimM* should be carefully selected as internal reference gene in studies involving cyanobacterial gene expression by qPCR. This is because it is necessary to verify the expression stability of multiple candidate reference genes under given experimental conditions and then choose the most stable reference to calibrate the relative expression of specific target genes [25]. Based on our results on the calibration of the target gene* kaiC *(Figure 4(b)), we confirmed that* rimM* was a suitable reference gene in* S. elongatus* PCC 7942 under NaCl stress. However, a double reference calibration by combining* rimM* with other suitable reference genes would further ensure the authenticity of the qPCR experimental results. In regard to the stability evaluation results of the nine candidate reference genes under the four simultaneous conditions, we found that the* prs *gene can be stably expressed under various stress conditions. Therefore,* prs *is an optimal reference gene for qPCR experiments in* S. elongatus* PCC 7942 under various environmental conditions such as NTC, HO, and HL (Table 4). The* prs* gene is one of the important housekeepers in cyanobacteria, and it encodes ribose phosphate pyrophosphate kinase, a key enzyme that is involved in the pentose phosphate pathway. These results also form an important physiological basis for the stable expression of this gene under various environmental stress conditions, as reported in this study.

In phylogenetic studies of prokaryotes, 16S ribosomal RNA is often selected as the target sequence for species-specific targeted amplification because of its conservative evolution and stable high expression [49, 50]. Interestingly, the 16S gene is also one of the most commonly used reference genes in qPCR experiments involving cyanobacteria [24, 29, 51–54]. The results of Pinto et al. showed that* 16S* effectively calibrates the expression of target genes in* Nostoc *sp. PCC 7120 and* Synecocystis *sp. PCC 6803 under various experimental conditions. However, in different species of cyanobacteria, the use of* 16S* as a reference gene for qPCR is controversial because the interpretation of results can be biased [25]. The results of present work also showed that the basal expression level of the* 16S *gene was too high, and its median Ct was 9.13 in qPCR (see Table 1). When* 16S *gene was used to calibrate specific low-abundance target genes, the very small median Ct of the target gene was very likely to artificially mask the differences in the relative expression of target genes between different sample groups. In addition, the comprehensive stability of the* 16S* gene under NaCl, HO, and HL stress was ranked 7th and 8th, respectively. These results showed that the expression of the 16S gene in cyanobacteria* S. elongatus *PCC 7942 under stress conditions was very unstable and was not the best choice as the reference gene in the qPCR reaction. This is due to the significant imbalance in the abundance between rRNA and mRNA molecules [55]. The different intracellular RNA degradation mechanisms also significantly affected the different half-lives of rRNA and mRNA [56]. On the other hand, the qPCR experiment itself usually requires the selection of a stably expressed gene having a similar Ct value as the reference gene [57]. Although the mRNA abundance of* rnpB* was also relatively high, the comprehensive ranking of the* rnpB *gene under all tested experimental conditions was always in the last two digits of the ranking, indicating that the expression of* rnpB* was unstable under almost all tested environmental conditions. This is consistent with the results of Pinto et al. Thus,* rnpB* is also not suitable for use as an internal reference gene in* S. elongatus* PCC 7942.

In summary, our results indicated that, in cyanobacteria* S. elongatus* PCC 7942,* secA* was the optimal reference gene in qPCR experiments under nonstress conditions, and* rnpA* and* ilvD* served as optimal reference genes under NaCl or HO conditions, respectively.* prs* could be used in a variety of experimental conditions for* S. elongatus *PCC 7942. However, reliable reference genes in qPCR experiments are always highly specific for individual environmental conditions. When performing qPCR in specific physiological or pathological conditions, it is important to first carefully evaluate and screen the reference genes that are most suitable for the preestablished experimental conditions as a necessary prerequisite for ensuring the credibility and reproducibility of the experiment [58]. The results of the current study showed that the expression stability of homologous housekeeping genes is not identical in different species of cyanobacteria or different sampling conditions. Previous evidence has shown that no housekeeping gene expression is stable under all environmental conditions [57]. In addition, if the reference gene stability evaluation results for a specific sample show that it is necessary to calibrate the Ct data of the target gene using two or more reference gene combinations together, the use of the two highest-ranking reference genes can simultaneously provide sufficient credibility and reproducibility for the qPCR experiment.

## 5. Conclusion

Based on geNorm, NormFinder, and BestKeeper gene expression stability analysis software programs, we studied the expression stability of nine candidate reference genes under four different experimental conditions in* S. elongatus* PCC 7942 and comprehensively evaluated their expression. The results of statistical analysis showed that the candidate reference genes* prs* and* secA *were the most stable in all samples tested,* secA *and* ppc *were most suitable for NTC stress,* rimM* and* rnpA* were most suitable for NaCl stress,* rnpA *and* ilvD* were most suitable for HO stress, and* prs* and* secA* were most suitable for HL stress.* rimM* was stably expressed only under specific conditions and should be carefully selected in future studies.* 16S* and* rnpB* were not suitable as reference genes. We anticipate that our results will provide a reliable experimental basis for the expression analysis of target genes under various stress conditions in* S. elongatus *PCC 7942 by the qPCR method.

## Figures and Tables

**Table 1:** Primer sequences and amplicon characteristics in the candidate reference genes.

Gene symbol	CYORF ID	Definition	Primer sequence (5′→3′)	Amplicon	Tm	E (%)	R^2^	Median Ct
			(Forward/Reverse)	length (bp)	(°C)			
*16S*	Synpcc 7942_R0052	16S ribosomal RNA	F: GCAAGCCTGACGGAGCAAC	180	58	96.3	0.997	9.13
			R: CGGACGCTTTACGCCCAAT					
*petB*	Synpcc 7942_2331	cytochrome b6	F: CTGATCCGCTCCATCCACC	296	58	90.4	0.999	20.88
			R: GTTGCCTGACCGACGCTTT					
*ppc*	Synpcc 7942_2252	phosphoenolpyruvate carboxylase	F: CCCTTGCCAGGACCAGATGA	219	62	96.7	0.999	25.83
			R: CGTCGGGTGAGCGGTGAAAA					
*ilvD*	Synpcc 7942_0626	dihydroxy-acid dehydratase	F: CGGCGCTGCGGCTCAATAT	128	58	97.4	0.999	24.09
			R: ATCATCGGCGGCGACAACC					
*prs*	Synpcc 7942_2113	ribose-phosphate pyrophosphokinase	F: TCTTGCCCTACTACGGTTACGC	102	58	91.1	0.999	24.70
			R: TCGCTCCAGCCTGAGTGATTAG					
*rnpA*	Synpcc 7942_1615	ribonuclease P protein component	F: CTCGTTGCCATCGACTGCG	130	62	94	0.996	26.93
			R: CGACTTTCGTTGACGGACTCTATTT					
*rnpB*	Synpcc 7942_R0036	RNA component of RNaseP	F: TACCGCCGATGGCCTGCTT	119	58	105.1	0.998	16.15
			R: TGTCCCTCCACCTTGCTCCC					
*secA*	Synpcc 7942_0289	preprotein translocase, SecA subunit	F: ACGACGGTCAGATTGCCGAGAT	204	59.1	97.6	0.998	24.97
			R: GCGACATTCCCTGCTGGATTAG					
*rimM*	Synpcc 7942_2259	16S rRNA processing protein RimM	F: TGATTGACCAAGCCAGCAGCAC	121	62	96.2	0.997	24.88
			R: GCCTCCACAAAGGGAATGTAGACC					

**Table 2:** Ranking of the candidate reference genes, as calculated by NormFinder software.

Rank		ALL	NTC	NaCl	HO	HL
1		*prs*	*secA*	*rimM*	*rnpA*	*rnpA*
	stability	0.307	0.176	0.144	0.096	0.136
2		*secA*	*ppc*	*ilvD*	*ilvD*	*prs*
	stability	0.309	0.210	0.199	0.160	0.185
3		*ilvD*	*rnpA*	*rnpA*	*ppc*	*secA*
	stability	0.467	0.272	0.226	0.195	0.208
4		*rnpA*	*prs*	*petB*	*prs*	*rimM*
	stability	0.494	0.280	0.256	0.221	0.234
5		*16S*	*16S*	*prs*	*secA*	*ppc*
	stability	0.524	0.296	0.279	0.268	0.254
6		*ppc*	*ilvD*	*ppc*	*petB*	*ilvD*
	stability	0.612	0.326	0.305	0.509	0.279
7		*rimM*	*petB*	*secA*	*rnpB*	*petB*
	stability	0.762	0.394	0.372	0.577	0.317
8		*petB*	*rnpB*	*16S*	*rimM*	*16S*
	stability	0.891	0.503	0.422	0.763	0.368
9		*rnpB*	*rimM*	*rnpB*	*16S*	*rnpB*
	stability	1.001	0.553	0.498	0.847	0.588

**Table 3:** Ranking of the candidate reference genes via the Pearson's correlation coefficient (r) and probability values (p), as calculated by BestKeeper software.

*Rank*	*ALL*			*NTC*			*NaCL*			*HO*			*HL*		
	Gene	r	p-value	Gene	r	p-value	Gene	r	p-value	Gene	r	p-value	Gene	r	p-value
1	*secA*	0.966	0.001	*16S*	0.937	0.001	*petB*	0.905	0.001	*rnpA*	0.972	0.001	*petB*	0.987	0.001
2	*prs*	0.956	0.001	*secA*	0.92	0.001	*rnpA*	0.885	0.001	*16S*	0.968	0.001	*prs*	0.983	0.001
3	*16S*	0.951	0.001	*rnpA*	0.881	0.001	*rimM*	0.882	0.001	*secA*	0.954	0.001	*rnpA*	0.976	0.001
4	*petB*	0.929	0.001	*ppc*	0.878	0.001	*16S*	0.87	0.001	*ilvD*	0.946	0.001	*secA*	0.968	0.001
5	*rnpA*	0.922	0.001	*prs*	0.862	0.001	*prs*	0.858	0.001	*ppc*	0.939	0.001	*rimM*	0.964	0.001
6	*ilvD*	0.855	0.001	*ilvD*	0.836	0.001	*ilvD*	0.857	0.001	*petB*	0.935	0.001	*ppc*	0.958	0.001
7	*ppc*	0.762	0.001	*petB*	0.721	0.005	*ppc*	0.811	0.001	*prs*	0.926	0.001	*ilvD*	0.935	0.001
8	*rimM*	0.681	0.001	*rnpB*	0.57	0.042	*secA*	0.669	0.012	*rnpB*	0.749	0.003	*16S*	0.924	0.001
9	*rnpB*	0.543	0.001	*rimM*	0.331	0.271	*rnpB*	0.477	0.099	*rimM*	0.506	0.077	*rnpB*	0.745	0.004

**Table 4:** Expression stability ranking of the nine candidate reference genes.

Method	1	2	3	4	5	6	7	8	9
(a) Ranking order under ALL sample (better–good–average)									
geNorm	*prs/secA*		*rnpA*	*16S*	*ilvD*	*ppc*	*petB*	*rimM*	*rnpB*
NormFinder	*prs*	*secA*	*ilvD*	*rnpA*	*16S*	*ppc*	*rimM*	*petB*	*rnpB*
BestKeeper	*secA*	*prs*	*16S*	*petB*	*rnpA*	*ilvD*	*ppc*	*rimM*	*rnpB*
Comprehensive ranking	*prs*	*secA*	*16S*	*rnpA*	*ilvD*	*petB*	*ppc*	*rimM*	*rnpB*
(b) Ranking order under NTC condition (better–good–average)									
geNorm	*prs/secA*		*ppc*	*ilvD*	*16S*	*rnpA*	*petB*	*rnpB*	*rimM*
NormFinder	*secA*	*ppc*	*rnpA*	*prs*	*16S*	*ilvD*	*petB*	*rnpB*	*rimM*
BestKeeper	*16S*	*secA*	*rnpA*	*ppc*	*prs*	*ilvD*	*petB*	*rnpB*	*rimM*
Comprehensive ranking	*secA*	*ppc*	*prs*	*16S*	*rnpA*	*ilvD*	*petB*	*rnpB*	*rimM*
(c) Ranking order under NaCl stress (better–good–average)									
geNorm	*ppc/prs*		*ilvD*	*rimM*	*rnpA*	*petB*	*secA*	*16S*	*rnpB*
NormFinder	*rimM*	*ilvD*	*rnpA*	*petB*	*prs*	*ppc*	*secA*	*16S*	*rnpB*
BestKeeper	*petB*	*rnpA*	*rimM*	*16S*	*prs*	*ilvD*	*ppc*	*secA*	*rnpB*
Comprehensive ranking	*rimM*	*rnpA*	*ilvD*	*petB*	*prs*	*ppc*	*16S*	*secA*	*rnpB*
(d) Ranking order under HO stress (better–good–average)									
geNorm	*ppc/prs*		*ilvD*	*rnpA*	*secA*	*petB*	*rnpB*	*rimM*	*16S*
NormFinder	*rnpA*	*ilvD*	*ppc*	*prs*	*secA*	*petB*	*rnpB*	*rimM*	*16S*
BestKeeper	*rnpA*	*16S*	*secA*	*ilvD*	*ppc*	*petB*	*prs*	*rnpB*	*rimM*
Comprehensive ranking	*rnpA*	*ilvD*	*ppc*	*prs*	*secA*	*petB*	*16S*	*rnpB*	*rimM*
(e) Ranking order under HL stress (better–good–average)									
geNorm	*prs/secA*		*petB*	*ppc*	*rnpA*	*ilvD*	*rimM*	*16S*	*rnpB*
NormFinder	*rnpA*	*prs*	*secA*	*rimM*	*ppc*	*ilvD*	*petB*	*16S*	*rnpB*
BestKeeper	*petB*	*prs*	*rnpA*	*secA*	*rimM*	*ppc*	*ilvD*	*16S*	*rnpB*
Comprehensive ranking	*prs*	*secA*	*petB*	*rnpA*	*ppc*	*rimM*	*ilvD*	*16S*	*rnpB*

**Table 5:** Comprehensive results of selected suitable reference genes based on geNorm, NormFinder, and BestKeeper.

Condition	Most		Least
ALL	*prs*	*secA*	*rnpB*
NTC	*secA*	*ppc*	*rimM*
NaCl	*rimM*	*rnpA*	*rnpB*
HO	*rnpA*	*ilvD*	*rimM*
HL	*prs*	*secA*	*rnpB*

## Data Availability

The dataset supporting the conclusions of this study is included within the article and supplementary information files.

## Abbreviations

*S. elongatus* PCC 7942: * Synechococcus elongatus* PCC 7942
qPCR: Quantitative real-time polymerase chain reaction
*16S*: 16S ribosomal RNA
ALL: All samples
NTC: No treatment control
NaCl: NaCl-treated group
HO: H_2_O_2_-treated group
HL: High light intensity-treated group.

## Supplementary Materials

Supplementary Materials Supplementary Figure S1: 1.5% agarose gel electrophoresis pattern of total RNA from time-course NTC cyanobacteria samples (no treatment control). Figure S2: the quantity and quality detection of total RNA from time-course NTC cyanobacteria samples (no treatment control) by a BioSpec-nano spectrophotometer. The ratio of A_260_/A_280_ and A_260_/A_230_ from each total RNA sample was also shown in graphs. (a) NTC-CT_0_. (b) NTC-CT_4_. (c) NTC-CT_8_. (d) NTC-CT_12_. (e) NTC-CT_16_. (f) NTC-CT_20_. (g) NTC-CT_24_. Figure S3: after amplification of each candidate reference gene PCR, the size of the expected fragment was visualized using agarose gel (1.5%) electrophoresis. M represents the 2000 bp DNA marker. Figure S4: melting curves of the nine candidate reference genes showed single peaks in qRT-PCR. Table S1: qPCR parameters for the amplification of* kaiC* under different experimental conditions.
