# Supplementary figures and images for: Stable Reference Gene Selection for RT-qPCR Analysis in Synechococcus elongatus PCC 7942 under Abiotic Stresses
Source: Biomed Res Int. 2019 Apr 21;2019:7630601. doi: 10.1155/2019/7630601 (PMC6500708; doi:10.1155/2019/7630601)

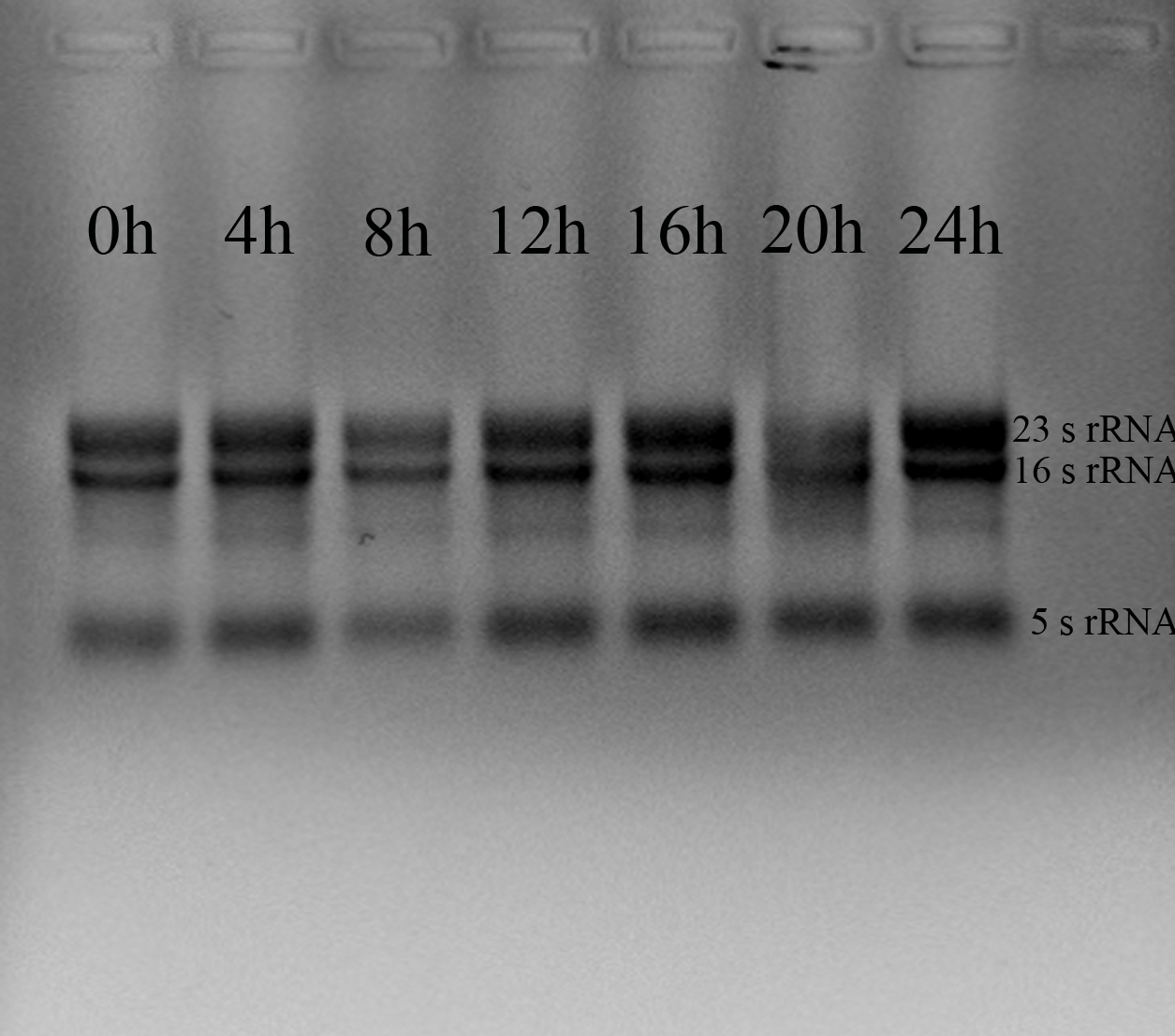

Supplement: Supplementary Materials — Supplementary Figure S1: 1.5% agarose gel electrophoresis pattern of total RNA from time-course NTC cyanobacteria samples (no treatment control). Figure S2: the quantity and quality detection of total RNA from time-course NTC cyanobacteria samples (no treatment control) by a BioSpec-nano spectrophotometer. The ratio of A260/A280 and A260/A230 from each total RNA sample was also shown in graphs. (a) NTC-CT0. (b) NTC-CT4. (c) NTC-CT8. (d) NTC-CT12. (e) NTC-CT16. (f) NTC-CT20. (g) NTC-CT24. Figure S3: after amplification of each candidate reference gene PCR, the size of the expected fragment was visualized using agarose gel (1.5%) electrophoresis. M represents the 2000 bp DNA marker. Figure S4: melting curves of the nine candidate reference genes showed single peaks in qRT-PCR. Table S1: qPCR parameters for the amplification of kaiC under different experimental conditions. [file 7630601.f1.zip › Figure S1.tif]

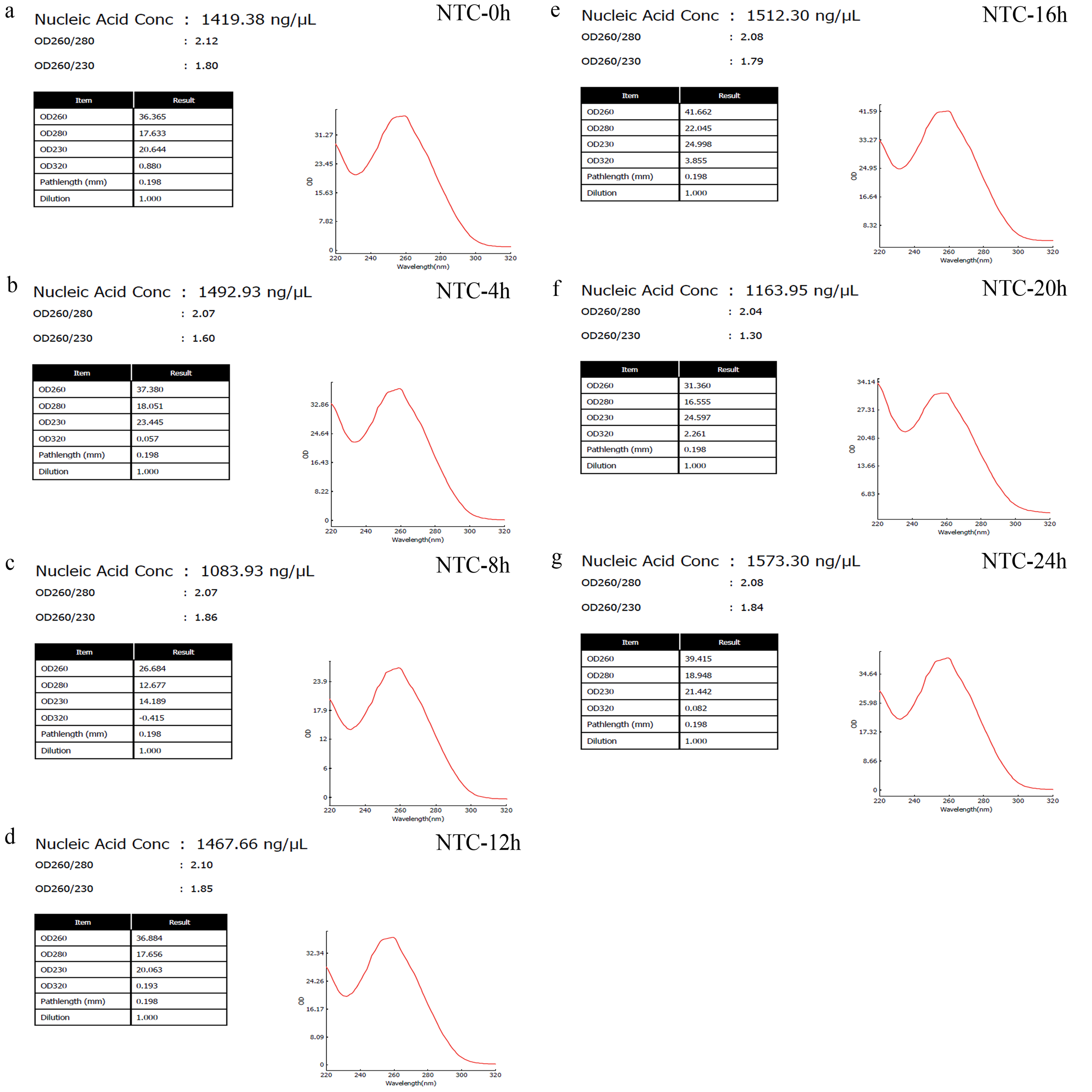

Supplement: Supplementary Materials — Supplementary Figure S1: 1.5% agarose gel electrophoresis pattern of total RNA from time-course NTC cyanobacteria samples (no treatment control). Figure S2: the quantity and quality detection of total RNA from time-course NTC cyanobacteria samples (no treatment control) by a BioSpec-nano spectrophotometer. The ratio of A260/A280 and A260/A230 from each total RNA sample was also shown in graphs. (a) NTC-CT0. (b) NTC-CT4. (c) NTC-CT8. (d) NTC-CT12. (e) NTC-CT16. (f) NTC-CT20. (g) NTC-CT24. Figure S3: after amplification of each candidate reference gene PCR, the size of the expected fragment was visualized using agarose gel (1.5%) electrophoresis. M represents the 2000 bp DNA marker. Figure S4: melting curves of the nine candidate reference genes showed single peaks in qRT-PCR. Table S1: qPCR parameters for the amplification of kaiC under different experimental conditions. [file 7630601.f1.zip › Figure S2.tif]

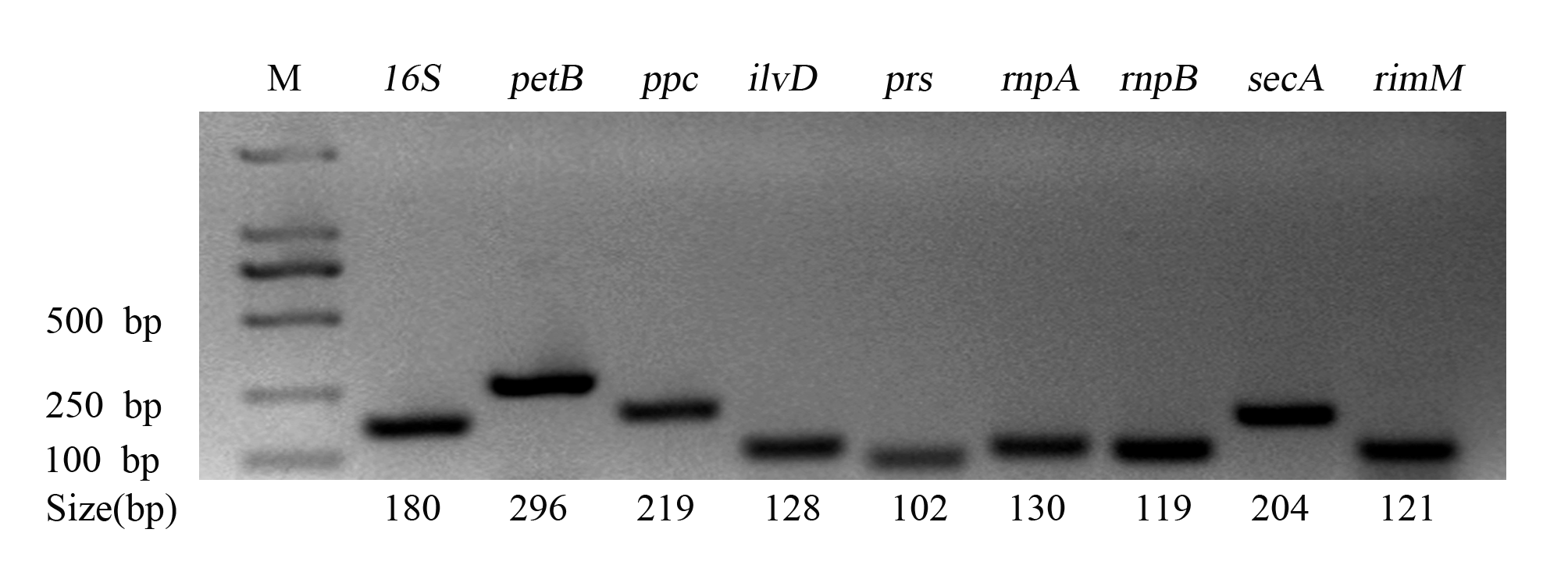

Supplement: Supplementary Materials — Supplementary Figure S1: 1.5% agarose gel electrophoresis pattern of total RNA from time-course NTC cyanobacteria samples (no treatment control). Figure S2: the quantity and quality detection of total RNA from time-course NTC cyanobacteria samples (no treatment control) by a BioSpec-nano spectrophotometer. The ratio of A260/A280 and A260/A230 from each total RNA sample was also shown in graphs. (a) NTC-CT0. (b) NTC-CT4. (c) NTC-CT8. (d) NTC-CT12. (e) NTC-CT16. (f) NTC-CT20. (g) NTC-CT24. Figure S3: after amplification of each candidate reference gene PCR, the size of the expected fragment was visualized using agarose gel (1.5%) electrophoresis. M represents the 2000 bp DNA marker. Figure S4: melting curves of the nine candidate reference genes showed single peaks in qRT-PCR. Table S1: qPCR parameters for the amplification of kaiC under different experimental conditions. [file 7630601.f1.zip › Figure S3.tif]

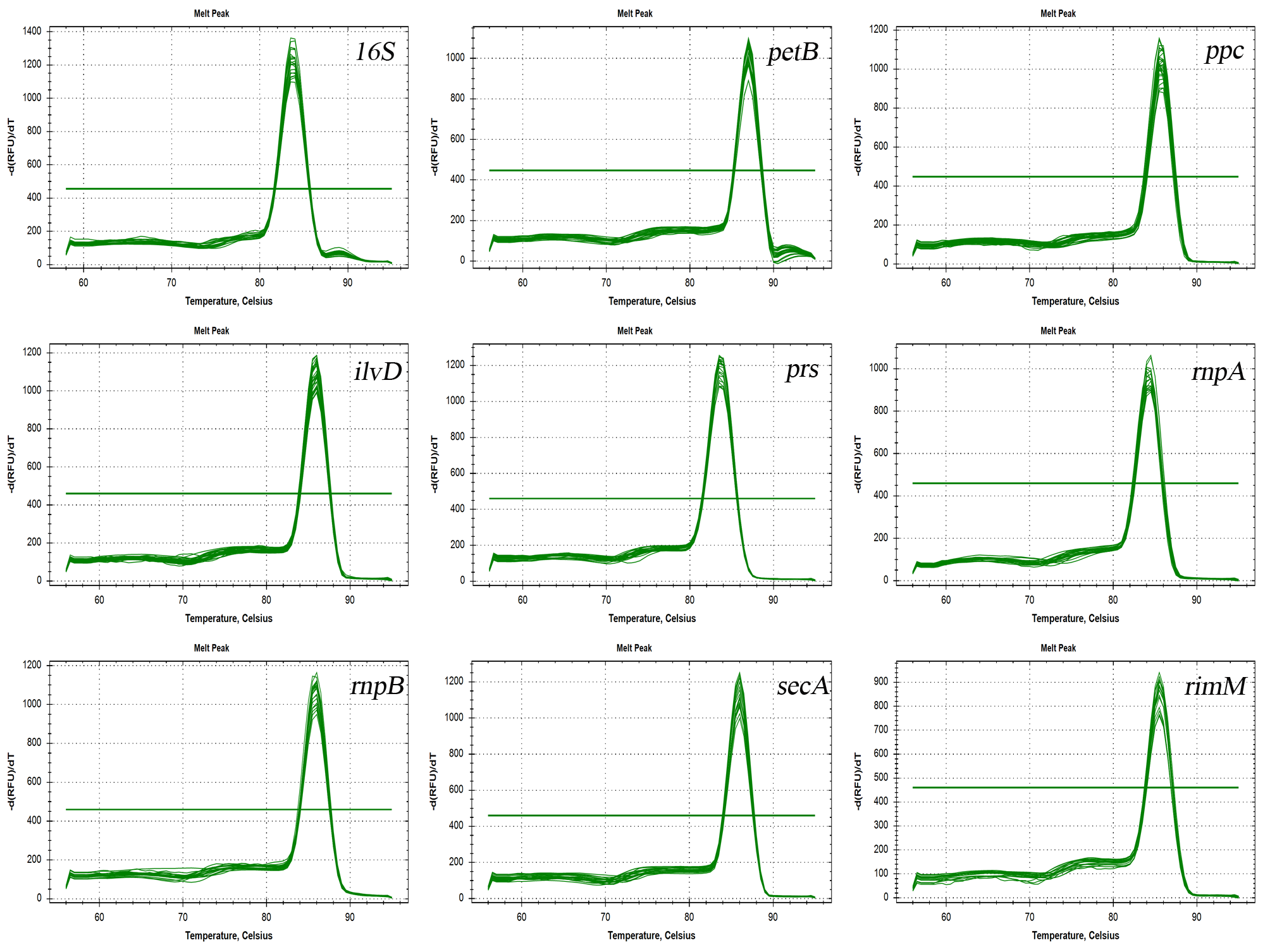

Supplement: Supplementary Materials — Supplementary Figure S1: 1.5% agarose gel electrophoresis pattern of total RNA from time-course NTC cyanobacteria samples (no treatment control). Figure S2: the quantity and quality detection of total RNA from time-course NTC cyanobacteria samples (no treatment control) by a BioSpec-nano spectrophotometer. The ratio of A260/A280 and A260/A230 from each total RNA sample was also shown in graphs. (a) NTC-CT0. (b) NTC-CT4. (c) NTC-CT8. (d) NTC-CT12. (e) NTC-CT16. (f) NTC-CT20. (g) NTC-CT24. Figure S3: after amplification of each candidate reference gene PCR, the size of the expected fragment was visualized using agarose gel (1.5%) electrophoresis. M represents the 2000 bp DNA marker. Figure S4: melting curves of the nine candidate reference genes showed single peaks in qRT-PCR. Table S1: qPCR parameters for the amplification of kaiC under different experimental conditions. [file 7630601.f1.zip › Figure S4.tif]
